# Review of real-world evidence of dual inhibition of VEGF-A and ANG-2 with faricimab in NAMD and DME

**DOI:** 10.1186/s40942-024-00525-9

**Published:** 2024-01-17

**Authors:** Fernando M Penha, Maliha Masud, Zoha A. Khanani, Mathew Thomas, Rodney D. Fong, Kyler Smith, Avishay Chand, Majid Khan, Greggory Gahn, Gustavo Barreto Melo, Arshad M. Khanani

**Affiliations:** 1 Fundação Universidade Regional de Blumenau, FURB, Blumenau, SC Brazil; 2 University of Nevada, Reno School of Medicine, Reno, NV USA; 3 Federal University of São Paulo, UNIFESP, São Paulo, SP Brazil; 4 Sierra Eye Associates, 950 Ryland St, Reno, NV 89502 USA

**Keywords:** Anti-VEGF-A, Angiopoietin 2, Neovascular age-related macular degeneration, Diabetic macular edema, Faricimab, Aflibercept, Brolucizumab, Ranibizumab, Bevacizumab, Intravitreal injection

## Abstract

Management of vitreoretinal disorders (e.g., neovascular age-related macular degeneration [nAMD] and diabetic macular edema [DME]) have assumed the standard therapy of lifelong anti-VEGF injections with drugs like aflibercept, brolucizumab, ranibizumab and bevacizumab. However, the burden imposed on patients is a major deterrent for continual therapy and recovery. Faricimab, a bispecific antibody, blocking both VEGF-A and Ang-2 molecules, produces a comparable functional and anatomical results, with less injections, significantly reducing patient burden. Visual acuity, safety, adverse effects, and anatomical outcomes are discussed in the pivotal clinical trials (YOSEMITE/RHINE and TENAYA/LUCERNE), and early data from real-world studies (TRUCKEE, TAHOE, FARWIDE-DME, FARETINA and others). In YOSEMITE and RHINE, faricimab demonstrated non-inferior vision gains, better anatomical outcomes compared to aflibercept every 8 weeks. Faricimab in the personalized treatment interval (PTI), after week 96, achieved 12-week interval in 78.1% of the patients and 16-week interval in 62.3%. TENAYA and LUCERNE reported comparable best corrected visual acuity (BCVA) improvement and better anatomic outcomes during head-to-head phase, parallel to aflibercept, at its 8-week treatment schedule. Faricimab in the PTI regimen, after week 96 achieved 12-week interval in 77.8% of the patients and 16-week interval in 63.1%. Safety of faricimab has been comparable to aflibercept in these pivotal trials. Real-world data supports the data from the pivotal studies regarding the efficacy and safety profile of faricimab in heterogenous real world patient population. Moreover, in previously treated patients, it also demonstrated a faster fluid resolution, good safety profile. Considering faricimab has demonstrated anatomic and durability benefit in the treatment of nAMD and DME, additional data from ongoing extension clinical trials, AVONELLE-X and RHONE-X will help understand longer term outcomes for patients treated with faricimab as well as patients switching from aflibercept to faricimab after finishing the pivotal trials. Longer term data from the real-world studies will also continue to contribute to our understanding of long-term efficacy, safety and durability in the real world patient population.

## Background

Neovascular age-related macular degeneration (nAMD) and diabetic macular edema (DME) are the main causes of visual loss and blindness in elderly and working-age adult patients. On its physiopathology it has significantly increased vitreous levels of vascular endothelial growth factor-A (VEGF-A), the main protein responsible for the increased vasculature permeability and angiogenesis, causing neovascularization, edema, and subsequent vision loss [1]. Targeting of proangiogenic cytokines allows for decreased expression of vitreoretinal angiogenesis and vascular permeability, slowing the progression of retinal degeneration [2]. Drugs such as bevacizumab, ranibizumab and aflibercept, inhibit VEGF-A with a good anatomical and functional improvement, with monthly or every other month intravitreal injections. Brolucizumab also blocks VEGF-A, with every 12-week regimen, however, due to safety concerns, its use is now very limited. Angiopoietin-2 (Ang-2) has also been demonstrated to be upregulated in patients with different retinal diseases [3]. Elevated levels of Ang-2 combined with VEGF-A leads to vascular instability, which causes vascular leakage, neovascularization, and inflammation [4]. Faricimab is a bispecific antibody, blocking both VEGF and Ang-2 molecules. This dual inhibition may induce more effective and durable fluid resolution and functional recovery in retinal vascular diseases. Here, we review the evidence from pivotal trials as well as real world studies about efficacy, safety, and durability of faricimab in patients with nAMD and DME (Tables 1 and 2).

**Table 1:** Pivotal studies - DME (YOSEMITE and RHINE), nAMD (TENAYA and LUCERNE)

Study	Disease	Study Design	Number of Patients (Faricimab)	Number of Patients (Aflibercept)	Key Findings
YOSEMITE (6)	DME	Randomized, double-blind, non-inferiority (Phase 3)	628	312	Faricimab non-inferior in visual gains and anatomical improvement compared to aflibercept every 8 weeks. Low rates of intraocular inflammation
RHINE (6)	DME	Randomized, double-blind, non-inferiority (Phase 3)	636	315	Faricimab non-inferior in visual gains and anatomical improvement compared to aflibercept every 8 weeks. Low rates of intraocular
TENAYA (13)	nAMD	Randomized, double-blind, non-inferiority (Phase 3)	334	337	Faricimab comparable to Aflibercept in visual gains and anatomical improvement at treatment intervals up to 16 weeks. Low rates of intraocular inflammation
LUCERNE (13)	nAMD	Randomized, double-blind, non-inferiority (Phase 3)	331	327	Faricimab comparable to Aflibercept in visual gains and anatomical improvement at treatment intervals up to 16 weeks. Low rates of intraocular inflammation

**Table 2:** Real-world studies

Study	Disease	Study Design	Number of Eyes (Faricimab)	Number of Patients (Faricimab)	Number of Injections	Key Findings
TAHOE (9)	DME	Investigator-initiated real-world study	181	136	756	After 3 injections, mean improvement of + 3.78 letters and reduction in CST of -45.28 µm. No reported intraocular inflammation
FARETINA-DME (9,10)	DME	Real-world study, using IRIS registry data	3961	2692		After 4 injections, stable vision in previously treated patients and a mean improvement of 3 letters in previously untreated patients
FARWIDE-DME (11)	DME	Observational, non-controlled study in the UK	2673	1921		After 4 injections, around 64% of previously treated eyes and 44% of untreated eyes achieved treatment intervals ≥ 8 weeks
Rush (12)	DME	Observational, non-controlled study	51	51		Aflibercept-resistant patients switched to faricimab; 39% with a dry macula at 12 months; -0.13 logMAR improvement
Kusuhara (13)	DME	Observational, non-controlled study	21	19		No statiscally signifcant improvement
TRUCKEE (15, 16)	nAMD	Investigator-initiated real-world study	2622	2212	11450	After the first injection, mean improvement of + 1.1 letters and reduction in CST of -31.3 µm. Two cases of idiopathic ocular inflammation reported
FARETINA-nAMD (17)	nAMD	Real-world study, using IRIS registry data	12.119	10551		After 4 injections, mean improvement of 0.5 letters in previously treated patients and 1.6 letters in untreated patients
FARWIDE-nAMD (18)	nAMD	Observational, non-controlled study in the UK	3564	2987		
Rush and Rush (19)	nAMD	Case-control study		27		Comparison with aflibercept. Higher improvement in the switch group from aflibercept to faricimab
Leung (20)	nAMD	Observational, non-controlled study in Atlanta, GA		190		After 3 injections, -0.06 logMAR improvement and − 25 µm CST reduction
Mukai (21)	nAMD	Observational, non-controlled study in Japan	63	61		82% of the eyes achieved a dry macula at month 3
Matsumoto (22)	nAMD	Observational, non-controlled study in Japan	40	38		LogMAR improvement of -0.11; dry macula in 79.5%; and complete polupoidal resolution in 61.1% of the eyes
Cheng (24)	nAMD	Observational, non-controlled study		13		
Stanga (23)	nAMD	Observational, non-controlled study		11		

## Main text

On January 28, 2022, the U.S. Food and Drug Administration (FDA) approved faricimab (Vabysmo, Genentech/Roche), for the treatment of retinal vascular diseases [1, 5]. Specifically, faricimab targets and inhibits VEGF-A and Ang-2, signaling proteins of two distinct pathways known to mediate vascular leakage and retinal neovascularization. The FDA approved faricimab for patients suffering from nAMD and DME, both of which are leading causes of blindness in elderly and working age adults, respectively. Through its innovative bispecific antibody activity and the release of phase 3 clinical trial results, faricimab has gained popular attention from the vitreoretinal community. However, safety concerns with new drugs in real-world scenario regarding this treatment remains. While phase 3 clinical trial data exist to support faricimab’s efficacy and safety at the time of this article’s publication, data from real world studies is important to efficacy and safety of faricimab in the real-world patient population.

## Diabetic macular edema (DME)

### Pivotal trials YOSEMITE and RHINE

Two randomized, double-masked, non-inferiority phase 3 trials, YOSEMITE and RHINE, evaluated the efficacy and safety of faricimab in the setting of DME to reduce treatment burden and optimize patient outcomes compared to the standard therapy aflibercept [6]. 1891 patients (YOSEMITE *n* = 940, RHINE *n* = 951) were randomized to intravitreal faricimab 6.0 mg every 8 weeks, faricimab 6.0 mg per personalized treatment interval (PTI), or aflibercept 2.0 mg every 8 weeks up to week 100. PTI is based on a protocol-driven dosing regimen, similarly to the so-called treat-and-extend regimen, wherein patients receive faricimab 6.0 mg every 4 weeks until central subfield thickness(CST) is less than 325 μm at/or after week 12, after which treatment intervals could be increased by 4 weeks (up to 16 weeks) or reduced by 4 or 8 weeks (down to 4 weeks) based on CST and best corrected visual acuity (BVCA). In both trials, Faricimab showed non-inferior vision gains and improved anatomical outcomes when compared to Aflibercept. Faricimab had a safety profile comparable to Aflibercept with low rates of intraocular inflammation: YOSEMITE: Faricimab every 8 weeks *n* = 5 (1.6%), Faricimab PTI *n* = 7 (2.2%), Aflibercept every 8 weeks *n* = 3 (1.0%) and RHINE: Faricimab every 8 weeks *n* = 3 (0.9%), Faricimab PTI *n* = 2 (0.6%), Aflibercept every 8 weeks *n* = 1 (0.3%). There were two cases of severe uveitis and one case of severe vitritis in YOSEMITE with Faricimab. All other adverse reactions were mild or moderate in severity. Overall, both YOSEMITE and RHINE achieved their endpoints and suggested that using a dual Ang-2 and VEGF-A bispecific antibody offered non-inferior vision gains and improved anatomical outcomes versus Aflibercept [6]. 

YOSEMITE and RHINE pooled data showed a comparable improvement in BCVA, + 11.2 letters (Faricimab PTI), + 11.2 letters (Faricimab Q8W) and + 10.5 letters (Aflibercept 2 mg Q8W) in the first year [6]. This improvement maintained up to year 2: + 10.8 letters (Faricimab PTI), + 10.4 letters (Faricimab Q8W) and + 10.3 letters (Aflibercept 2 mg Q8W) [7]. Anatomical improvement was also achieved with central subfield thickness (CST) resolution: − 200.9 micra (Faricimab Q8W), − 192.4 micra (Faricimab PTI), − 170.2 micra (Aflibercept 2 mg Q8W) in year one and were maintained in year 2 [7]. 

### Real-world studies

#### The TAHOE trial

The TAHOE trial is a collaborative, investigator initiated real-world study with both naïve and previously treated DME patients, with at least one intravitreal injection of faricimab in the United States [8]. 44.7% of the patients were previously treated with other intravitreal agents, such as aflibercept (39 eyes, 21.5%), bevacizumab (26 eyes, 14.4%), ranibizumab (15 eyes, 8.3%), and dexamethasone implant (1 eye, 0.56%). Only 10 eyes (5.5%) were treatment-naïve patients.

At the recent EURETINA 2023 annual scientific meeting, Nielsen et al. presented the very first real-world data on faricimab for DME in the TAHOE study. In the 140 study eyes with follow-up data after the first faricimab injection, visual acuity (measured in ETDRS letters) improved by + 2.16 letters. Anatomic outcomes measured by CST improved by -36.16 μm. After 3 faricimab injections (94 eyes) patients improved + 3.78 letters and reduced CST by -45.28 μm. Regarding the safety profile, after 756 injections (181 eyes), there were no cases of intraocular inflammation, endophthalmitis, vasculitis, or retinal artery occlusion reported in TAHOE to date. The study is currently ongoing, and more data is expected to be presented in the near future [8]. 

#### FARETINA-DME

FARETINA-DME is a retrospective real world study using data from the IRIS registry. During the ASRS 41st 2023 annual meeting Borkar et al. presented the data from February to September 2022 seeking for patients treated with faricimab for DME. Patients receiving ≥ 4 faricimab injections were included in the analyses of injection intervals and best documented visual acuity (BDVA) [9]. 

A total of 3961 eyes, representing 2692 patients, underwent treatment with faricimab for diabetic macular edema. On average, these eyes received 2.6 (standard deviation 1.3) injections over a period of 55.1 (standard deviation 47.2) days of follow-up. Among these eyes, 497 (12.5%) had not previously received anti-VEGF treatment, while 3464 (87.5%) had been treated before. Among those who had received prior treatment, 75.5% had previously been treated with aflibercept [9]. After 4 initial faricimab injections vision was stable in previously treated eyes; however, it improved in treatment-naïve patients, 3 letters on average. Interval treatment extension of 6 weeks or more was achieved after 1–2 injections by 61.8% of previously treated eyes and 61.5% of treatment-naïve eyes [9, 10]. 

#### FARWIDE-DME

The FARWIDE-DME is an observational, noncontrolled study in the UK that enrolled patients treated with at least one faricimab injection both naïve and previously treated diabetic macular edema. The source data was recorded from 21 National Health System sites in the United Kingdom (Medisoft EMR system) [11]. 

At the recent EURETINA 2023 annual scientific meeting, Bailey C. et al. presented the very first data from FARWIDE-DME from June 2022 to June 2023, with 1921 patients and 2673 eyes. Most eyes were previously treated (1721 eyes, 64.4%) and 952 eyes (35.6%) were treatment naïve eyes. The mean treatment interval extends to 8 weeks after the 4th injection in treatment-naïve eyes. After the 4th injection approximately 64% of the naïve eyes and 44% of previously treated eyes were on ≥ 8wk intervals [11]. 

#### Other real world data

Ryan B. Rush published a retrospective interventional case series with intravitreal faricimab in patients with aflibercept-resistant diabetic macular edema. This cohort included patients that received at least 8 injections in a 12 month or 4 injections in a 6-month period, and despite of that, remained with CST above 320 microns and visual acuity between 20/25 and 20/200 [12]. Patients received at least three loading doses of faricimab, and then monthly injection until no fluid was noticed to start treat and extend approach. A total of 51 eyes of 51 subjects were analyzed. There were 39.2% (20/51) of patients who reached a treatment interval of ≥ 8 weeks and had a fluid-free macula on OCT at 12 months. The CMT on OCT of the patient population reduced from 400.2 (385.3– 415.3) microns at baseline to 340.6 (324.3–356.9) microns at 12 months (*p* < 0.01). There were 21.6% (11/51) of patients who improved ≥ 3 lines of Snellen visual acuity at 12 months. The visual acuity of the overall study population improved from 0.60 (0.54–0.66) logMAR (Snellen 20/80) at baseline to 0.47 (0.41–0.53) logMAR (Snellen 20/59) at 12 months (*p* < 0.01) [12]. There were no safety data presented in this paper.

Kusuhara et al. reported a short-term outcome of intravitreal faricimab in DME patients. A total of 21 eyes from 19 patients were treated. The mean number of faricimab injections was 1.6 ± 0.8 during the mean follow-up time of 5.5 months. There was an improvement in vision acuity, but it was not significantly compared to baseline. CST significantly decreased from baseline to 1 month (*p* = 0.001) but did not reach a significant level over 6 months (*p* = 0.070). No serious safety concerns were observed in this cohort [13]. 

## Neovascular age-related macular degeneration (nAMD)

### Pivotal trials TENAYA and LUCERNE

Similarly, the TENAYA and LUCERNE studies are two randomized, double-masked, non-inferiority trials comparing visual acuity, anatomic, and safety outcomes of faricimab on 8-, 12-, and 16-week treatment regiments to the standard of care aflibercept at 8-week intervals in the setting of nAMD [14]. The most recent data on week 112 of treatment suggest that faricimab at 16-week treatments is comparable to aflibercept at 8-week treatments. Mean BCVA changes from aggregated 8-week, 12-week, and 16-week faricimab treatment schedules at weeks 104–112 of treatment were 3.7 letters vs. 3.3 letters for aflibercept 8-week treatments in TENAYA and 5.0 vs. 5.2 in LUCERNE. These findings indicate that vision gains from baseline were comparable between faricimab up to 16-week intervals and aflibercept 8-week intervals. (Khanani 2021) CST reduction was − 146.5 μm with faricimab vs. -146.2 μm with aflibercept in TENAYA and − 150.3 μm vs. -141.6 μm in LUCERNE. In assessing the proportion of patients who gained or maintained ≥ 15 BCVA letters, faricimab was comparable to aflibercept in both TENAYA and LUCERNE. All study efficacy endpoints at 112 weeks of treatment mimicked study endpoint findings at 48 weeks with 74.1% of faricimab participants on ≥ 12 weeks dosing in TENAYA and 81.2% in LUCERNE.

At the 112-week evaluation point, the incidence of ocular serious adverse events in the study eye was low and comparable between faricimab and aflibercept groups in both TENAYA (faricimab *n* = 14 [4.2%] vs. aflibercept *n* = 13 [3.9%]) and LUCERNE (faricimab *n* = 15 [4.5%] vs. aflibercept *n* = 16 [4.9%]). Intraocular inflammatory rates were faricimab *n* = 11 (3.3%) vs. aflibercept *n* = 5 (1.5%) in TENAYA, and faricimab *n* = 9 (2.7%) vs. aflibercept *n* = 10 (3.1%) at treatment week 112. All new events in the faricimab treatment arm of each study since week 48 of the endpoint analysis were reported as nonserious and mild/moderate in severity. Thus far, faricimab has been well tolerated and shows a strong durability signal with roughly 80% of participants on ≥ 12-week dosing schedules. Faricimab also currently shows comparable BCVA improvement and CST reduction compared to aflibercept at 8-week treatment schedules [14]. 

### Real-world studies

#### The TRUCKEE trial

The TRUCKEE trial is collaborative, investigator initiated real-world study with both naïve and previously treated nAMD patients, with at least one intravitreal injection of faricimab in the United States [15]. Most patients (89.6%) were previously treated with other anti-VEGF agents, aflibercept (237 eyes, 63%), ranibizumab (58 eyes, 15.4%), brolucizumab (26 eyes, 6.9%) and bevacizumab (16 eyes, 4.3%). Only 39 eyes (10.4%) were treatment-naïve patients.

In the 376 study eyes with follow-up data after the first faricimab injection, visual acuity improved by + 1.1 letters. Anatomic outcomes measured by CST and retinal pigment epithelial detachment (PED) height both improved by -31.3 μm and − 58.9 μm, respectively. More notably, this study reported 2 cases of idiopathic ocular inflammation (IOI), with no cases of vasculitis, or retinal artery occlusion. Among these 2 cases of IOI, one was a case of infectious endophthalmitis, in which visual acuity returned to baseline following treatment, and the second a mild anterior chamber inflammation treated with topical steroids [15]. 

At the recent EURETINA annual scientific meeting (2023), Almeida et al. presented more recent data from the TRUCKEE trial. A total of 2622 eyes received at least one faricimab injection and 397 eyes at least 6 injections [16]. In this smaller subset of previously treated patients with longer follow-up, we see meaningful and statistically significant improvements functional and anatomically. In this cohort with at least 6 injections in patients switched from any anti-VEGF (397 eyes), the visual acuity improved in + 0.47 letters and CST improved in − 24.08 μm and PED. In this cohort 11,450 were performed. The IOI rate was 0.02%, endophthalmitis 0.04%, with no cases of retinal vasculitis or artery occlusion.

The TRUCKEE study is continuing to gather data, specifically looking at durability and long-term safety profile as patients receive multiple injections; further findings are expected to be presented in future meetings.

#### FARETINA-AMD

FARETINA-AMD is a retrospective real world study using data from the IRIS registry. During the ARVO 2023 annual meeting, Borkar et al. presented the data from February to August 2022 seeking for patients treated with faricimab for nAMD. Patients receiving ≥ 4 faricimab injections were included in the analyses of injection intervals and BDVA [17]. 

A total of 12,119 eyes, representing 10,551 patients, underwent treatment with Faricimab for neovascular age-related macular degeneration (nAMD). On average, these eyes received 2.6 (standard deviation 1.3) injections over a period of 55.1 (standard deviation 47.2) days of follow-up. Among these eyes, 1,633 (13.5%) had not previously received anti-VEGF treatment, while 10,486 (86.5%) had been treated before. Among those who had received prior treatment, 55.1% had previously been treated with aflibercept [17]. 

At the outset of the faricimab treatment, nearly half of the eyes, both treatment-naïve (46%) and previously treated (47%), had BDVA of 20/40 or better. A total of 426 (26.1%) treatment-naïve eyes and 2,438 (23.3%) previously treated eyes received four or more injections. After receiving four injections, the mean change in BDVA was 0.5 (SD 8.9) letters for previously treated eyes and 1.6 (SD 8.9) letters for treatment-naïve eyes. Furthermore, 236 (55.4%) of the treatment-naïve eyes and 1,542 (63.3%) of the previously treated eyes experienced at least one “extended” injection interval within the initial four injections.

#### FARWIDE-nAMD

The FARWIDE-nAMD is an observational, noncontrolled study in the UK that enrolled patients treated with at least one faricimab injection both naïve and previously treated diabetic macular edema. The source data was recorded from 14 National Health System sites in the United Kingdom (Medisoft EMR system) [18]. 

At the recent ASRS 2023 annual scientific meeting, Patel C. et al. presented the very first data from FARWIDE-nAMD from June 2022 to February 2023, with 2987 patients and 3564 eyes. Most eyes were previously treated (275 eyes, 77.2%) and 814 eyes (22.8%) were treatment naïve. Most previously treated patients received aflibercept injections (85.9%). After 5 injections visual appeared stable in previously treated eyes while statistically significant and VA gains were observed in treatment-naïve eyes [18]. 

#### Other real-world data

Rush and Rush published a retrospective case-control study with intravitreal faricimab in patients with aflibercept-resistant neovascular AMD [19]. This study included 55 patients, which 28 switched to faricimab and received 3 injections within 4 months. 27 patients continued with intravitreal injections of aflibercept, also receiving 3 injections within 4 months. The primary endpoint was the percentage of patients achieving CST less then 300 microns, without observable intraretinal or subretinal fluid at the end of month 4. Patients switching to faricimab led to significant improvements in vision and anatomically. 39.3% vs. 7.4% met the anatomical endpoint with fluid resolution after the initial 4 months, respectively with faricimab and aflibercept. Similarly, 35.7% in the faricimab group and 7.4% in the aflibercept group improved 2 or more visual acuity lines from baseline. There were no safety data presented in this paper.

This single center retrospective study from Atlanta, GA, USA, reported 190 treatment-resistant nAMD eyes who received at least 3 intravitreal faricimab injections with at least 3 months of follow-up. The average BCVA improved from 0.33 ± 0.32 logMAR to 0.27 ± 0.32 logMAR. The CST improved from 312 ± 87 μm to 287 ± 71 μm. Fluid resolution at last visit (week 12) was achieved by 24% of the patients. No patients developed idiopathic intraocular inflammation [20]. 

Mukai et al. reported 61 patients (63 eyes) with no previous injections that were treated with three injections of faricimab for neovascular AMD. In this Japanese cohort, a total of 82% of the eyes achieved a dry macula at month 3, defined as the absence of intraretinal or subretinal fluid. Moreover, a complete regression of polypoidal lesions was observed in 52% of eyes with PCV. Regarding safety profile, two eyes developed retinal pigment epithelium tears, but no other ocular or systemic complications were observed [21]. 

In another Japanese report, Matsumoto et al. reported 38 eyes from 40 treatment naïve patients with nAMD that received intravitreal injections of faricimab. Vision significantly improved from 0.33 ± 0.41 to 0.22 ± 0.36 logMAR by week 16. Regarding anatomical results at week 16, foveal thickness reduced significantly by 105 μm, CST by 22 μm and dry macula was achieved in 79.5% of the eyes (31 out of 39). After the loading phase, complete regression of polypoidal lesions was observed in 61.1% of eyes (11 out of 18) with polypoidal lesions. A safety issue was observed: one eye (2.5%) experienced vitritis without loss of vision at week 16 [22]. 

There are also some small case series reported by Cheng et al. (13 eyes), Stanga et al. (11 eyes) with similar anatomical and functional improvements after faricimab injections with no safety concerns [23, 24]. 

## Lessons from the real word data

With the shared goal of decreasing patient’s treatment burden, multiple anti-VEGF drugs have emerged with innovative modalities designed to achieve increased efficacy, durability and treatment interval while maintaining a relatively safe treatment profile in managing retinal disease [1–4, 25]. 

Current pivotal faricimab has provided strong data regarding the safety, durability and efficacy of this new drug in naïve DME and nAMD patients. The YOSEMITE/RHINE trials and the TENAYA/LUCERNE trials, along with preliminary findings from real-world studies such as TRUCKEE, TAHOE, FARWIDE-DME, and FARETINA, indicate a reduction in treatment burden associated with faricimab. This is observed across various pertinent indicators that significantly alleviate the overall treatment burden for patients. Recognizing this reduction is crucial in the context of long-term disease management and patient adherence. However, as observed in the real-world studies, most patients that have received this drug are switch patients. It is very important to observe if the anatomic and durability benefit seen in the pivotal clinical trials also is confirm in the real-world scenario in long term. Regarding the safety profile of faricimab, recent real-world data provides additional confirmation of its efficacy, showing no emergence of new safety signals within a diverse patient population.

1. Several real-world data presented so far shows anatomic and durability benefit of faricimab in tough to treat as well as naïve patients with no new safety signals. Outside of specific inclusion exclusion criteria and a homogenous clinical trial patient population, in clinical practice, we have a very heterogeneous patient population. Some of these patients may have autoimmune or other clinical uncontrolled diseases and may be positive for anti-drug antibody (ADA) from other intravitreal agents used previously. Those characteristics may alter the clinical response and safety of the drug. However, the real-world study data to date, safety remains very similar to what was observed in pivotal trials.
2. Recently, Genentech/Roche updated the faricimab label to include the risk of retinal vasculitis. According to the press release, as of the August 2023, with 1.5 million vials dosed globally, the estimated reporting rate of retinal vasculitis with occlusion is 0.06/10,000 injections (for retinal vasculitis with or without occlusion: 0.17/10,000 injections). Per the updated US product information, the adverse reactions of retinal vasculitis with or without retinal vascular occlusion were included in the label. Since these reactions are reported voluntarily from a population of uncertain size, it is not possible to reliably estimate their frequency or establish a causal relationship to drug exposure. More information about these cases will be available in the near future [26].

Based on what was presented in the paper regarding the real-world studies it is possible to make some observations:

1. A single injection of faricimab results in rapid improvement in all anatomic parameters in treatment-naïve and previously treated patients with nAMD and DME [8–11, 27, 28].
2. Most patients switching from aflibercept 2 mg to faricimab experience reductions in CST/IRF/SRF/PED, along with stable or improved vision with the potential for extended treatment intervals in both nAMD and DME [9–11, 27, 28].
3. Notal Oct Analyzer (NOA) fluid quantification sub-study has indicated that faricimab offers advantages in improving fluid status of patients with nAMD [29].
4. Majority but not all faricimab-treated patients will improve after one injection and loading maybe necessary.
5. In high-need switch patients, loading may continue to improve anatomy.
6. Faricimab may potentially lead to fast resolution of sub-retinal hemorrhage and sub-retinal hyperreflective material while minimizing fibrosis - further data is required to confirm this observation [30].
7. A subset of treatment-naïve nAMD patients may be extended longer than q16 weeks with faricimab.
8. Faricimab demonstrates favorable safety profile, with rates of inflammation and endophthalmitis comparable to other commonly employed treatments.

## Future directions

Anti-VEGF agents have been the gold standard for the treatment of retinal diseases since the FDA approval of Ranibizumab in 2006. However, advancements in drug delivery technology, have made it possible to discover other potential targets and modalities to avoid progressive vision loss. The development of the PDS and other drug delivery strategies aims to improve the paradigm of lifelong repeat injections [31, 32]. PDS is a permanent intraocular refillable implant loaded with ranibizumab. With PDS, continuous release of the drug can be achieved for up to 6 months before requiring refilling [31]. 

Gene therapies present exciting progress in the development of viral vector therapeutics for AMD. Unlike monoclonal antibody and receptor fusion protein therapy approaches, gene therapies for AMD target native ocular tissue to deliver a one-time genetic payload capable of sustained anti-VEGF protein production [33]. Ultimately, gene therapies for AMD aim to reduce the treatment burden currently in place for AMD with a single injection. ADVM-022 (NCT03748784), RGX-314 (NCT05407636; NCT04704921; NCT04514653; NCT04567550), GT-005 (NCT05481827; NCT04437368; NCT04566445; NCT03846193), and HMR59 (NCT03144999; NCT03585556) are gene therapies currently undergoing clinical trials to evaluate safety and efficacy in the treatment of wet and dry AMD, as well as diabetic macular edema. However, cost remains a limiting factor in access to gene therapies with further work needed to optimize tissue specificity, vector production, and transgene expression.

## Conclusions

We have excellent treatments for patients with nAMD and DME but they require frequent injections. As a field, we continue to look for new treatment options with the goal of decreasing treatment burden and improving visual outcomes. Faricimab is a novel drug that provides anatomical and functional improvement, with greater treatment intervals up to 16 weeks, reducing patient burden. Real world studies reviewed in this paper show superior anatomic benefit of faricimab in treatment naïve and previously treated patients in heterogenous patient population. The safety seen in these studies is also comparable to what was seen in the pivotal trial. It is important to continue to generate long term data for faricimab in real world patient population to establish efficacy, safety and durability seen with dual inhibition of VEGF-A and Ang2.

## Data Availability

Available upon request.

## Abbreviations

FDA: Food and drug association
VEGF: A-vascular endothelial growth factor
nAMD: neovascular age-related macular degeneration
DME: diabetic macular edema
PTI: personalized treatment interval
IOP: intraocular pressure elevation
CST: central subfield thickness
BCVA: best-corrected visual acuity
BDVA: best-documented visual acuity
ASRS: American Society of Retina Specialists
PED: pigment epithelial detachment
IOI: idiopathic orbital inflammation
FAB: fragment antigen-binding (region)
IRIS: intelligent research in sight (registry)
PDS: port delivery system
